# Asymmetry of the Structural Brain Connectome in Healthy Older Adults

**DOI:** 10.3389/fpsyt.2013.00186

**Published:** 2014-01-09

**Authors:** Leonardo Bonilha, Travis Nesland, Chris Rorden, Julius Fridriksson

**Affiliations:** ^1^Department of Neurosciences, Medical University of South Carolina, Charleston, SC, USA; ^2^Department of Psychology, University of South Carolina, Columbia, SC, USA; ^3^Department of Communication Sciences and Disorders, University of South Carolina, Columbia, SC, USA

**Keywords:** asymmetry, structural connectome, connectivity, diffusion tensor imaging, aging

## Abstract

**Background:** It is now possible to map neural connections *in vivo* across the whole brain (i.e., the brain connectome). This is a promising development in neuroscience since many health and disease processes are believed to arise from the architecture of neural networks.

**Objective:** To describe the normal range of hemispheric asymmetry in structural connectivity in healthy older adults.

**Materials and Methods:** We obtained high-resolution structural magnetic resonance images (MRI) from 17 healthy older adults. For each subject, the brain connectome was reconstructed by parcelating the probabilistic map of gray matter into anatomically defined regions of interested (ROIs). White matter fiber tractography was reconstructed from diffusion tensor imaging and streamlines connecting gray matter ROIs were computed. Asymmetry indices were calculated regarding ROI connectivity (representing the sum of connectivity weight of each cortical ROI) and for regional white matter links. All asymmetry measures were compared to a normal distribution with mean = 0 through one-sample *t*-tests.

**Results:** Leftward cortical ROI asymmetry was observed in medial temporal, dorsolateral frontal, and occipital regions. Rightward cortical ROI asymmetry was observed in middle temporal and orbito-frontal regions. Link-wise asymmetry revealed stronger connections in the left hemisphere between the medial temporal, anterior, and posterior peri-Sylvian and occipito-temporal regions. Rightward link asymmetry was observed in lateral temporal, parietal, and dorsolateral frontal connections.

**Conclusion:** We postulate that asymmetry of specific connections may be related to functional hemispheric organization. This study may provide reference for future studies evaluating the architecture of the connectome in health and disease processes in older individuals.

## Introduction

With recent advances in structural neuroimaging, it is now possible to track medium and large-scale pathways of white matter fibers and to construct the map of neural connectivity across the entire brain (the brain connectome) (1, 2). The brain connectome constitutes a promising development in neuropsychiatry since many physiological and pathological processes are believed to affect the architecture of neural networks (3–6). For example, normal cognitive development spanning from childhood to senior years is traditionally believed to be associated with maturation of neural networks supporting cognitive domains such as attention (7), memory (8), language (9), and executive function (10). Similarly, neurological diseases including epilepsy and dementia are associated with pathological rearrangements or impoverishment of normal networks at a systems level (11, 12). Furthermore, psychiatric diseases such as schizophrenia (13), bipolar disorder (14), and addiction (15) are related to reinforcements of pathological networks.

In order to better understand how different biological processes affect the normal connectome, it is important to accurately characterize the connectome organization in healthy individuals. Specifically, it is important to define the degree of individual variability and hemispheric asymmetry that can be expected from the normal population.

Since hemispheric asymmetry is abnormal in neuropsychiatric diseases such as schizophrenia (16), bipolar disorder (17, 18), and depression (19), in this study we aimed to describe the patterns of hemispheric asymmetry and individual variability of the brain connectome obtained from a cohort of healthy senior individuals. We employed high-resolution magnetic resonance images (MRI) to reconstruct structural brain connectivity based upon white matter pathways linking anatomically defined cortical regions of interest (ROIs). We aimed to describe the group-wise distribution of asymmetries involving cortical connectivity and white matter pathways.

## Materials and Methods

### Subjects

We studied 17 right-handed healthy subjects (mean age 53 years, SD = 7 years, range = 40–76) who were recruited from the local community. None of the subjects had a history of neurological, psychiatric, or chronic medical illnesses. All patients signed an informed consent to participate in this study. The Institutional Review Board at the University of South Carolina approved this study.

### Image acquisition

All subjects underwent MRI scanning at a 3T Siemens Trio equipped with a 12-channel head coil located at the University of South Carolina, yielding: (1) T1-weighed images (3D MP-RAGE, TR = 2250 ms, TE = 4.15 ms, 256 × 256 matrix, 256 mm × 256 mm FOV, parallel imaging GRAPPA = 2, 80 reference lines, TA = 377 s); and (2) Diffusion weighted images (dMRI)-EPI scan (30-directions with *b* = 1000 s/mm^2^ and *b* = 2000 s/mm^2^, TR = 6100 ms, TE = 101 ms, 82 × 82 matrix, 222 mm × 222 mm FOV, parallel imaging GRAPPA = 2, 80, 45 contiguous 2.7 mm axial slices, TA = 390 s).

### Image pre-processing

The construction of the connectome involved two parallels pre-processing steps, namely, the segmentation of the cerebral cortex into multiple anatomical ROIs and reconstruction of white matter fibers. These steps are explained below:

### Segmentation of the cerebral cortex

T1-weighted MR images were converted into NIfTI format utilizing the dcm2nii tool from the MRIcron software package (20). Images in native space were non-linearly normalized into standard MNI space using the Clinical Toolbox (21) employing unified segmentation-normalization routines as part of the software Statistical Parametric Mapping (SPM8). Of note, the Clinical Toolbox was particularly designed to accurately quantify tissue volumes in seniors and older adults (21).

This step yielded probabilistic maps of gray and white matter in MNI space.

Next, the base *b* = 0 T2-weighted dMRI volume was linearly transformed to standard space utilizing a boundary-based registration approach (22). This step was performed using FMRIB’s Linear Image Registration Tool (FLIRT), as part of FMRIB Software Library (FSL) (23). The registration parameters were then used to transform the Automated Anatomical Labeling (AAL) atlas (24) and the white and gray matter probabilistic maps onto the dMRI space. Once in dMRI space, a map of cortical regions segmented according to AAL was obtained by overlaying the registered AAL atlas onto the registered probabilistic gray matter map. The intersection between these images (including only voxels with a probability greater than 50% of being gray matter) represented the segmented cortical map. A list describing all ROIs used in this study can be observed in Table A1 in Appendix.

### White matter fiber reconstruction

Extraction of diffusion gradients was performed with dcm2nii (20). The dMRI volumes were aligned to the *b* = 0 dMRI image using the FSL FLIRT tool (23). In diffusion space, whole brain tractography was reconstructed with the software Diffusion Toolkit (25) according to the following parameters: (1) angle threshold = 45°, (2) inclusion mask derived from the average of diffusion weighted signal and from the white matter probabilistic map registered to dMRI space, (3) FACT propagation algorithm, (4) spline filter.

### Calculation of the connectome

From each patient, the path of each tractography streamline was assessed. All streamlines were seeded in white matter, and the end-points of each streamline were computed. Streamlines with end-points within ROIs were counted as links between these ROIs. Streamlines with end-points outside ROIs were discarded. After all streamlines were assessed, the result was a weighted connectivity matrix **A**, where the entry **A**_ij_ represented the number of streamlines connecting regions *i* and *j* (i.e., the weighted link between *i* and *j*). Note that only direct links between regions *i* and *j* were included in the link A_ij_.

Finally, each link weight was corrected based on the surface of the connected ROIs and the distance between the ROIs, as proposed by Hagmann et al., where the link weight is inversely proportional to the sum of the linked ROI surfaces and fiber length, in order to account for tractography bias related to size of connected ROIs and distance traveled by the streamline (2).

### Connectome asymmetry

#### Asymmetry was evaluated for ROIs and for links

Regions of interested asymmetry was calculated by assessing the sum of link weights connecting an ROI, in comparison with the homologous ROI in the contralateral hemisphere. The connectivity of each ROI was computed without discrimination regarding the opposite end of the streamline. For example, when computing the connectivity of the left hippocampus, all possible connections of the left hippocampus were computed, including connections to ROIs in the ipsilateral and contralateral hemispheres. Connections of the left hippocampus to itself or to the contralateral hippocampus were discarded.

For each ROI, cortical connectivity asymmetry was calculated according to the following asymmetry index (AI) = (R-L)/[(R + L)/2]; where L represents the connectivity of the ROI in the left hemisphere and R represents the connectivity of the ROI in the homologous ROI in the right hemisphere. A one-sample *t*-test was performed to evaluate whether the distribution of ROI asymmetries across all subjects was statistically different than a distribution with mean = 0. This step was performed for each ROI. Note that ROI asymmetry represents the sum of connections to an ROI, irrespective to which other ROI is being linked to that ROI. Thus, ROI asymmetry should be interpreted in the context of link-wise asymmetry.

Link-wise hemispheric asymmetry was calculated by assessing the difference in weight for each link, according to the same asymmetry index AI = (R-L)/[(R + L)/2]; where L represents the link between ROIs within the left hemisphere and R represents the link between the homologous ROIs within the right hemisphere. Note that inter-hemispheric connections were excluded from this calculation. Cerebellar links were also excluded. Only supratentorial links within the same hemisphere were assessed. A one-sample *t*-test was performed to evaluate whether the distribution of asymmetries for each link across subjects was statistically different than a distribution with mean = 0.

## Results

**Figure 1:**
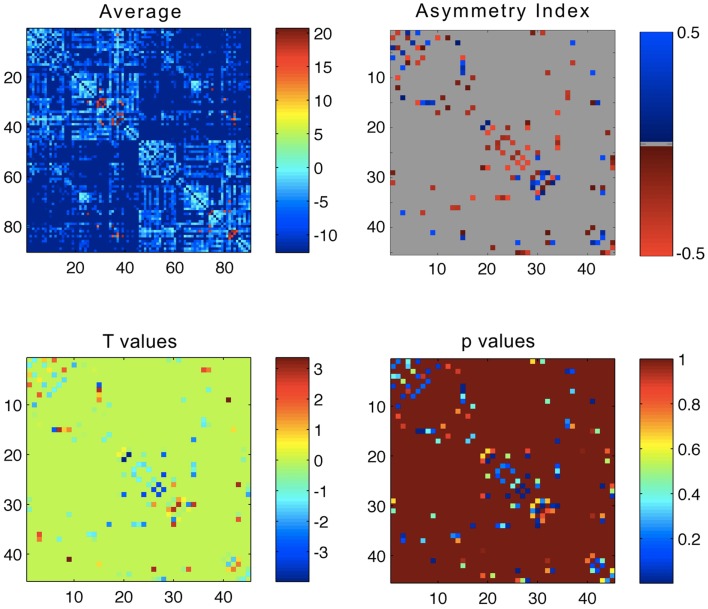
**Upper left panel: average connectivity matrix from all subjects – the scale bar represents log(connectivity weight)**. Upper right panel – link-wise average asymmetry index. Lower left panel – *T* values from a one-sample *t*-test evaluating the differences between the asymmetry indices and a distribution with mean = 0. Lower right panel – *p* values from the one-sample *t*-test. For all plots, the *x*- and *y*-axis represent ROIs numbered from 1 to 90 in accordance, where ROIs 1–45 are located in the left hemisphere, and 46–90 in the right hemisphere. The legend for ROI numbering can be seen in Table A1 in Appendix.

The average connectivity matrix is demonstrated in Figure 1. The ROIs are numbered in accordance with the glossary from Table A1 in Appendix. Briefly, regions 1–45 represent ROIs located in the left hemisphere, whilst regions 46–90 represent ROIs located in the right hemisphere. As such, within the average connectivity matrix with 90 × 90 entries, the upper left quadrant demonstrates links between ROIs in the left hemisphere, and the lower right quadrant demonstrates links between ROIs within the right hemisphere. The right upper and the left lower quadrants of the connectivity matrix represent reciprocal left to right connections. Since connections are not directed (i.e., the strength of connectivity from region *i* to *j* is the same as the connectivity from region *j* to *i*), the matrix is symmetrical along its main diagonal. As expected, links within the same hemisphere exhibited a higher weight compared with connectivity links between different hemispheres.

The average link-wise hemispheric asymmetry is also demonstrated in Figure 1. This matrix only includes 45 × 45 entries, where each cell represents the asymmetry index for each link. In order to avoid false estimations of asymmetry in links that were tracked only in a few subjects, we only included links that were tracked in greater than 75% of subjects (i.e., at least 13/17 subjects). Each matrix cell represents the asymmetry index for the connection between the ROI listed in the column and the ipsilateral ROI listed in the row. The ROIs are numbered in accordance with Table A1 in Appendix (from 1 to 45, regardless of side).

**Figure 2:**
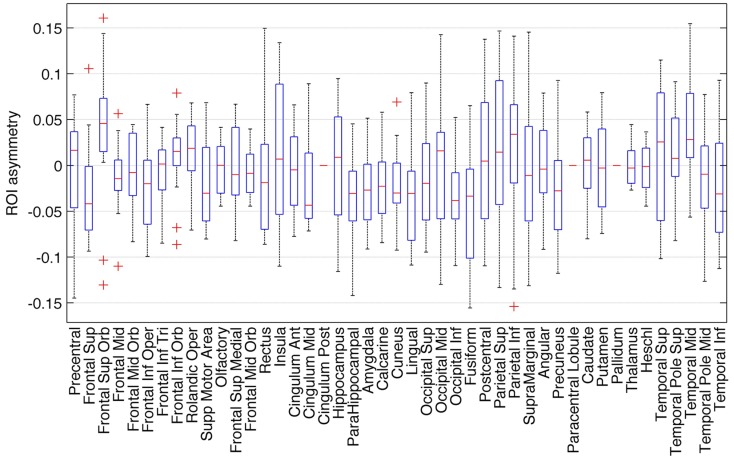
**Box-plot demonstrating the distribution of asymmetry indices for all ROIs**. Positive asymmetry indices represent a leftward asymmetry.

The distribution of ROI asymmetries is shown in Figure 2. The box-plot demonstrates the range of asymmetries across all subjects for each ROI. As expected, there were no regions of extreme asymmetry. A one-sample *t*-test revealed that among all possible 45 ROIs, a significant leftward asymmetry was noted on the inferior occipital region, fusiform gyrus, lingual gyrus, parahippocampal gyrus, amygdala, inferior frontal operculum, superior frontal gyrus, and mid cingulate gyrus. Conversely, a rightward asymmetry was noted for the middle temporal gyrus and superior frontal orbital region. These results are shown in Table 1.

**Table 1:** **Asymmetry of cortical ROI global connectivity**.

Region	Asymmetry index	*p*	*T*
**LEFTWARD ASYMMETRY**			
Occipital inf	−0.0381	0.0023	−3.6133
Fusiform	−0.0486	0.0065	−3.1304
Lingual	−0.0376	0.0067	−3.1154
Parahippocampal	−0.0320	0.0073	−3.0732
Amygdala	−0.0237	0.0241	−2.4916
Frontal inf oper	−0.0257	0.0361	−2.2873
Frontal sup	−0.0280	0.0464	−2.1587
Cingulum mid	−0.0232	0.0488	−2.1321
**RIGHTWARD ASYMMETRY**			
Temporal mid	0.0368	0.0100	2.9201
Frontal sup orb	0.0445	0.0283	2.4112

*Only ROIs with a *t*-test *p* value less than 0.05 are shown*.

Link-wise asymmetry was observed toward both hemispheres. A significant leftward asymmetry was noted on the following reciprocal connections: amydgala to parahippocampal gyrus; middle to inferior occipital regions; fusiform to lingual gyri; fusiform to occipital inferior gyrus; insula to inferior frontal opercular region; fusiform to parahippocampal gyrus; precuneus to lingual gyrus; angular to superior parietal region; precuneus to mid cinculate gyrus. Conversely, a significant rightward asymmetry was noted on the following reciprocal connections: superior temporal gyrus to rolandic opercular region; middle temporal gyrus to middle occipital region; inferior to superior parietal regions; insula to inferior frontal triangularis region; precuneus to superior parietal region. These results are summarized in Table 2.

**Table 2:** **Asymmetry of cortical links**.

Link between		Asymmetry index	*P*	*T*
**LEFTWARD ASYMMETRY**				
Amygdala	Parahippocampal	−0.8411	0.0011	−3.9764
Occipital inf	Occipital mid	−0.8139	0.0080	−3.0298
Fusiform	Lingual	−0.5496	0.0087	−2.9857
Fusiform	Occipital inf	−0.7223	0.0113	−2.8617
Insula	Frontal inf oper	−0.7726	0.0156	−2.7069
Fusiform	Parahippocampal	−0.4685	0.0239	−2.4952
Precuneus	Lingual	−0.6617	0.0406	−2.2281
Angular	Parietal sup	−0.6378	0.0421	−2.2092
Precuneus	Cingulum mid	−0.5340	0.0453	−2.1715
**RIGHTWARD ASYMMETRY**				
Temporal sup	Rolandic oper	0.6482	0.0040	3.3592
Temporal mid	Occipital mid	0.6424	0.0098	2.9310
Parietal inf	Parietal sup	0.6973	0.0113	2.8605
Insula	Frontal inf tri	0.4912	0.0129	2.7959
Precuneus	Parietal sup	0.6894	0.0157	2.7016

*Only links with a *t*-test *p* value less than 0.05 are shown*.

**Figure 3:**
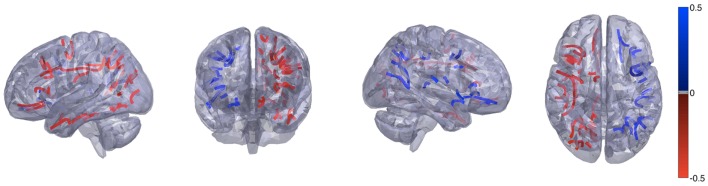
**Anatomical demonstration of the location of links with an absolute asymmetry index greater than 0.5**. Links in red exhibit a leftward asymmetry, while links in blue demonstrate a rightward asymmetry.

These results are demonstrated in Figure 1, which shows the distribution of *T* scores and *p* values for all links, represented in 45 × 45 matrices, where each matrix cell demonstrates the *T* value or the *p* value for the asymmetry distribution for the connection between the row ROI and the column ROI. Figure 3 demonstrates the anatomical distribution of links with an absolute mean asymmetry higher than 0.5. The three-dimensional anatomical reconstruction of regional links was defined based on an in-house developed atlas of anatomical connectivity involving all 90 ROIs used in this study. The location of travel of all streamlines connecting each possible pair of ROIs was defined based on the spatial distribution of center points (or “centroid”) of serial transverse sections across the white matter streamlines corresponding to each link, through an in-house modified version of the methods described by Garyfallidis et al (26). The centroids for each link were connected to define the main pathway of streamline travel. This step was repeated for all possible links. The utility of this approach was exclusively for anatomical visualization of the results, as demonstrated in Figure 3.

## Discussion

In this study, we investigated the individual variability of hemispheric asymmetry in structural connectivity in healthy senior individuals. By reconstructing the structural connectome from each subject, and by assessing the distribution of asymmetry indices related to cortical ROI connectivity and link weight, we observed that, within a sample composed of healthy older adults, there was mild but noticeable hemispheric asymmetry in structural connectivity.

We observed a more prominent leftward asymmetry in cortical connectivity, i.e., a larger number of regions demonstrated a higher degree of connectivity in the left hemisphere. Specifically, cortical ROIs located in the occipital lobe, medial temporal, dorsolateral and medial frontal lobe and cingulate exhibited a higher weight of connectivity in the left hemisphere. Conversely, fewer cortical regions demonstrated a rightward asymmetry, with the middle temporal gyrus and the orbito-frontal regions exhibiting a significantly higher cortical connectivity on the right hemisphere.

In turn, we also observed regional hemispheric asymmetry in relationship with the strength of connectivity between specific ROIs. In accordance with the previous observation about global cortical connectivity, a leftward asymmetry was also more commonly observed among links. Interestingly, a leftward asymmetry was observed on peri-Sylvian and medial temporal – occipital regions. Conversely, a rightward asymmetry was noted on lateral temporal – frontal – occipital and parietal links. While did not test the relationship between these links and cognitive performance, it is possible to speculate that some lateral asymmetry may be related to functional specialization of some of these connections. For instance, leftward asymmetry may be associated with dominant hemisphere language processing involving verbal memory (parahippocampal – amydala connections) (27), phonological processing (insula – frontal operculum connections) (28), and semantic retrieval (29) (angular gyrus – superior parietal connections). Conversely, a rightward asymmetry may be observed in networks associated with visual-spatial processing (30) (intraparietal connections, precuneus-parietal connections).

It should be noted that we adopted a liberal statistical threshold (i.e., the level of statistical significance from the one-sample *t*-tests was not corrected based on multiple comparisons). We adopted a liberal threshold since we expected that the degree of asymmetry exhibited by our population would be mild, and given the number of multiple comparisons, a more stringent threshold would preclude the evaluation of the locations with a higher degree of asymmetry. Nonetheless, given our sample size, it is possible that some of our observed asymmetries may be related to sample bias and may constitute false positives. For this reason, we recommend the interpretation of the results from this manuscript in this context. The asymmetry index is possibly a better representation of the magnitude of asymmetry, rather than over-emphasizing the importance of links or ROIs with *p* < 0.05.

The results reported in this study should help the contextual evaluation of other connectome studies applied to health and disease. We demonstrated the range of asymmetry in a small cohort of normal older adults with the purpose of providing a reference for future studies evaluating processes that affect neural network organization. Thus, future studies should also be interpreted with special attention to specific characteristics of the demographics from the population studied.

## Conflict of Interest Statement

The authors declare that the research was conducted in the absence of any commercial or financial relationships that could be construed as a potential conflict of interest.

## Appendix

**Table A1:** **Legends for ROI numbering**.

Number	Side	ROI	Number	Side	ROI
1	Left	Precentral	46	Right	Precentral
2	Left	Frontal sup	47	Right	Frontal sup
3	Left	Frontal sup orb	48	Right	Frontal sup orb
4	Left	Frontal mid	49	Right	Frontal mid
5	Left	Frontal mid orb	50	Right	Frontal mid orb
6	Left	Frontal inf oper	51	Right	Frontal inf oper
7	Left	Frontal inf tri	52	Right	Frontal inf tri
8	Left	Frontal inf orb	53	Right	Frontal inf orb
9	Left	Rolandic oper	54	Right	Rolandic oper
10	Left	Supp motor area	55	Right	Supp motor area
11	Left	Olfactory	56	Right	Olfactory
12	Left	Frontal sup medial	57	Right	Frontal sup medial
13	Left	Frontal mid orb	58	Right	Frontal mid orb
14	Left	Rectus	59	Right	Rectus
15	Left	Insula	60	Right	Insula
16	Left	Cingulum ant	61	Right	Cingulum ant
17	Left	Cingulum mid	62	Right	Cingulum mid
18	Left	Cingulum post	63	Right	Cingulum post
19	Left	Hippocampus	64	Right	Hippocampus
20	Left	Parahippocampal	65	Right	Parahippocampal
21	Left	Amygdala	66	Right	Amygdala
22	Left	Calcarine	67	Right	Calcarine
23	Left	Cuneus	68	Right	Cuneus
24	Left	Lingual	69	Right	Lingual
25	Left	Occipital sup	70	Right	Occipital sup
26	Left	Occipital mid	71	Right	Occipital mid
27	Left	Occipital inf	72	Right	Occipital Inf
28	Left	Fusiform	73	Right	Fusiform
29	Left	Postcentral	74	Right	Postcentral
30	Left	Parietal sup	75	Right	Parietal sup
31	Left	Parietal inf	76	Right	Parietal inf
32	Left	Supramarginal	77	Right	Supramarginal
33	Left	Angular	78	Right	Angular
34	Left	Precuneus	79	Right	Precuneus
35	Left	Paracentral lobule	80	Right	Paracentral lobule
36	Left	Caudate	81	Right	Caudate
37	Left	Putamen	82	Right	Putamen
38	Left	Pallidum	83	Right	Pallidum
39	Left	Thalamus	84	Right	Thalamus
40	Left	Heschl	85	Right	Heschl
41	Left	Temporal sup	86	Right	Temporal sup
42	Left	Temporal pole sup	87	Right	Temporal pole sup
43	Left	Temporal mid	88	Right	Temporal mid
44	Left	Temporal pole mid	89	Right	Temporal pole mid
45	Left	Temporal inf	90	Right	Temporal inf
